# The Effects of Biopolymer Encapsulation on Total Lipids and Cholesterol in Egg Yolk during *in Vitro* Human Digestion

**DOI:** 10.3390/ijms140816333

**Published:** 2013-08-07

**Authors:** Sun-Jin Hur, Young-Chan Kim, Inwook Choi, Si-Kyung Lee

**Affiliations:** 1 Department of Bioresources and Food Science, Konkuk University, 120 Neungdong-ro, Gwangjin-gu, Seoul 143-701, Korea; E-Mail: lesikyung@konkuk.ac.kr; 2 Korea Food Research Institute, 1201-62 Anyangpangyo-ro, Bundang-gu, Gyeonggi-do 463-746, Korea; E-Mails: yckim@kfri.re.kr (Y.-C.K.); choiw@kfri.re.kr (I.C.)

**Keywords:** egg yolk, cholesterol, biopolymers, *in vitro* human digestion, lipase activity

## Abstract

The purpose of this study was to examine the effect of biopolymer encapsulation on the digestion of total lipids and cholesterol in egg yolk using an *in vitro* human digestion model. Egg yolks were encapsulated with 1% cellulose, pectin, or chitosan. The samples were then passed through an *in vitro* human digestion model that simulated the composition of mouth saliva, stomach acid, and the intestinal juice of the small intestine by using a dialysis tubing system. The change in digestion of total lipids was monitored by confocal fluorescence microscopy. The digestion rate of total lipids and cholesterol in all egg yolk samples dramatically increased after *in vitro* human digestion. The digestion rate of total lipids and cholesterol in egg yolks encapsulated with chitosan or pectin was reduced compared to the digestion rate of total lipids and cholesterol in other egg yolk samples. Egg yolks encapsulated with pectin or chitosan had lower free fatty acid content, and lipid oxidation values than samples without biopolymer encapsulation. Moreover, the lipase activity decreased, after *in vitro* digestion, in egg yolks encapsulated with biopolymers. These results improve our understanding of the effects of digestion on total lipids and cholesterol in egg yolk within the gastrointestinal tract.

## 1. Introduction

Eggs are an important part of human diet [1] and contain high amounts of cholesterol. The average egg contains 213 mg of cholesterol [2], which is approximately twice the amount of cholesterol that is found in butter and freeze-dried meat products and about 5–10 times more cholesterol than that found in most dairy products [3]. In general, an increase in the concentration of blood cholesterol is widely recognized as a risk factor for coronary artery disease [4]. Hence, the consumption of table eggs has decreased in many developed countries during the last 4 decades due to these perceptions about cholesterol [1]. However, some studies [5,6] have reported that healthy individuals can consume 1 egg per day without affecting their blood cholesterol levels or increasing their risk of cardiovascular disease.

Numerous studies have demonstrated the ability of biopolymers such as chitosan, pectin, guar gum, xanthan gum, or modified starch to function as dietary fibers by lowering blood cholesterol concentrations and reducing lipid absorption [7]. In a previous study, Lairon *et al.* reported that dietary fibers can alter the breakup and coalescence of lipid droplets in the stomach and small intestine, thereby altering the surface area of emulsified lipids exposed to digestive enzymes [8]. Hur *et al.* reported that dietary fibers from various sources can bind to bile acids as well as mixed micelle components; they also explained particle disruption of the micellization process, which leads to reduced micellar solubilization of lipids [7]. For instance, cationic chitosan can bind to the surface of anionic lipid droplets, which are stabilized by bile salt or phospholipids, and reduce lipase activity by preventing contact between lipase and emulsified lipid substrates [9]. However, how biopolymer encapsulation influences the digestion of total lipids and cholesterol in egg yolk remains to be elucidated. Thus, the purpose of this study was to determine the effects of biopolymer encapsulation on the digestion of total lipids and cholesterol present in egg yolk using an *in vitro* human digestion model.

## 2. Results

### 2.1. Digestion Rates of Total Lipids and Cholesterol

**Figure 1:**
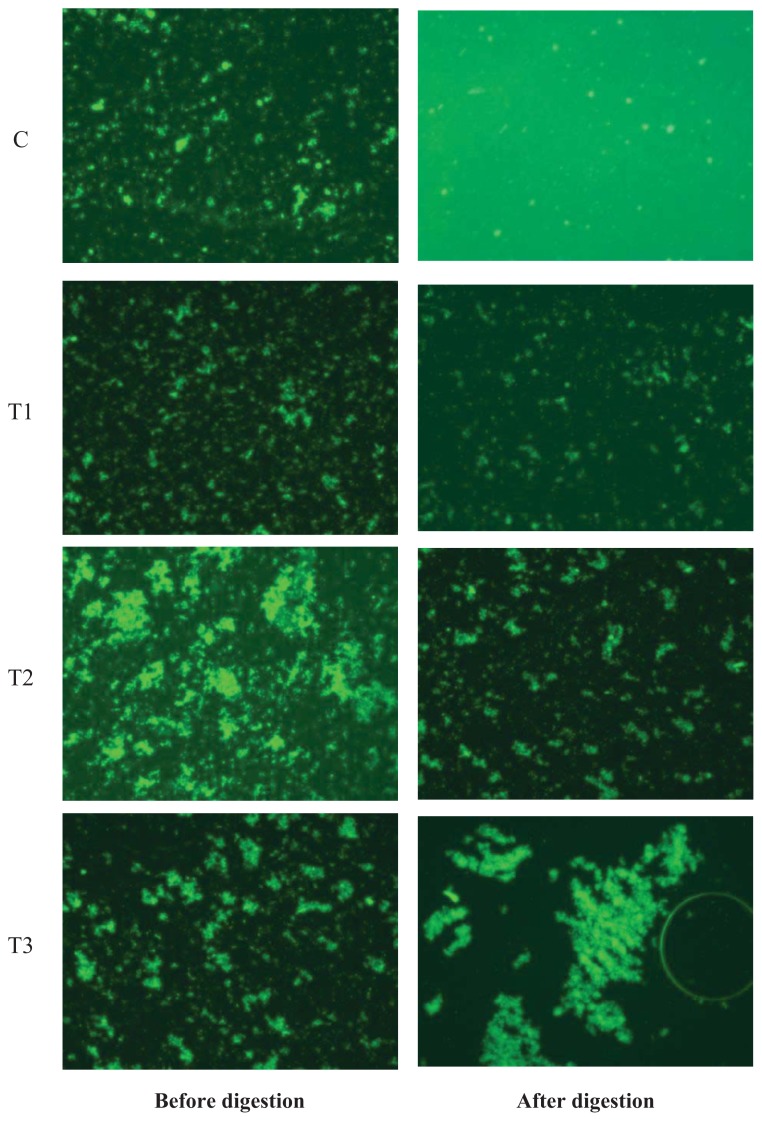
Effect of biopolymers encapsulation on the digestion of total lipids in egg using an *in vitro* human digestion model. C: no encapsulation; T1: encapsulation with cellulose; T2: encapsulation with pectin; T3: encapsulation with chitosan.

Representative confocal images of egg yolks before and after *in vitro* human digestion are presented in Figure 1.

**Figure 2:**
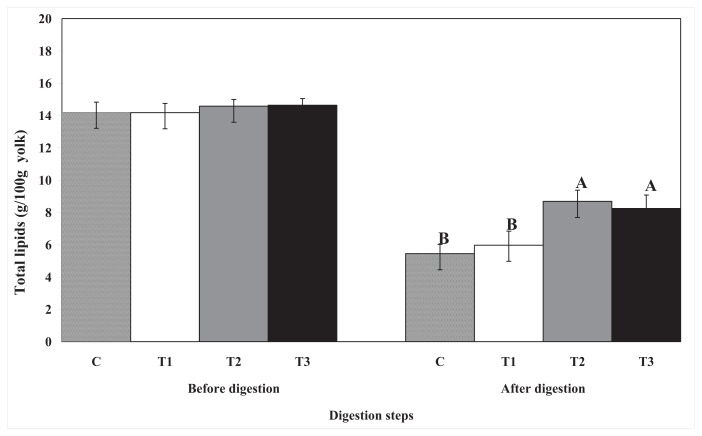
The effect of biopolymer encapsulation on total lipid content in egg yolk during *in vitro* human digestion. C: no encapsulation; T1: encapsulation with cellulose; T2: encapsulation with pectin; T3: encapsulation with chitosan.

Prior to digestion, lipid droplets appear to be covered by chitosan and pectin, whereas cellulose did not have a covering effect on the egg yolk (Figure 1). The size of the lipid droplets in the control and cellulose-encapsulated samples were much smaller than the size of droplets in the chitosan- or pectin-encapsulated samples. The lipid digestion in the control and cellulose-encapsulated samples was higher than that in the chitosan- or pectin-encapsulated samples (Figure 2).

**Figure 3:**
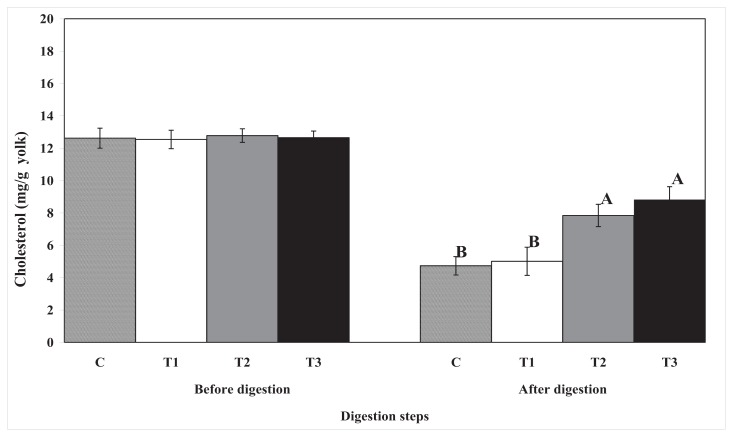
The effect of biopolymer encapsulation on the cholesterol content in egg yolk during *in vitro* human digestion. C: no encapsulation; T1: encapsulation with cellulose; T2: encapsulation with pectin; T3: encapsulation with chitosan. The results of before digestion are total cholesterol content, and results of after digestion are undigested cholesterol content.

The cholesterol content decreased in all egg yolk samples after *in vitro* simulated human digestion (Figure 3). After *in vitro* human digestion, the undigested cholesterol content was higher in the egg yolks encapsulated with chitosan or pectin than in the control or cellulose-encapsulated samples. These results suggest that during *in vitro* human digestion, total lipid and cholesterol digestion can be reduced by encapsulating egg yolks with pectin or chitosan. However, the reduction in digestion rate of total lipids and cholesterol in pectin- and chitosan-encapsulated egg yolks was similar.

### 2.2. Free Fatty Acid

**Figure 4:**
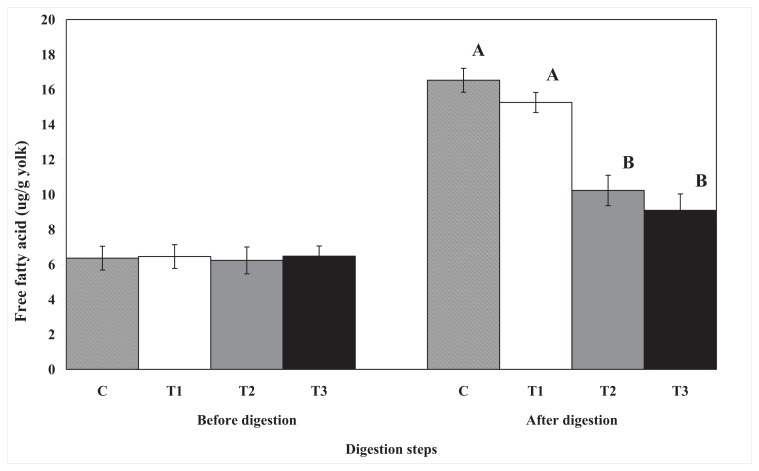
The effect of biopolymer encapsulation on free fatty acid content in egg yolk during *in vitro* human digestion. C: no encapsulation; T1: encapsulation with cellulose; T2: encapsulation with pectin; T3: encapsulation with chitosan.

The effect of biopolymer encapsulation on the digestion of total lipids and cholesterol was determined by measuring the level of free fatty acids before and after *in vitro* human digestion (Figure 4). The amount of free fatty acid dramatically increased after *in vitro* human digestion in all egg yolk samples. The amount of free fatty acids after *in vitro* human digestion was lower in egg yolk samples encapsulated with pectin or chitosan than in the other samples, whereas the samples encapsulated with cellulose had the same free fatty acid content as the control samples. The amount of free fatty acids in chitosan- or pectin-encapsulated egg yolks did not differ. The fatty acid composition of palmitic acid and oleic acid changed after *in vitro* human digestion in all egg yolk samples. However, the levels of total saturated and unsaturated fatty acids were not significantly different in any of the egg yolk samples (data are not shown). The release of free fatty acid indicates that lipids are hydrolyzed by digestive enzymes or bile salts during *in vitro* human digestion. In this regard, the decrease in the free fatty acid content, by biopolymer encapsulation, after *in vitro* human digestion may be closely related to the decrease in the digestion rate of total lipids and cholesterol.

### 2.3. Lipid Oxidation

**Figure 5:**
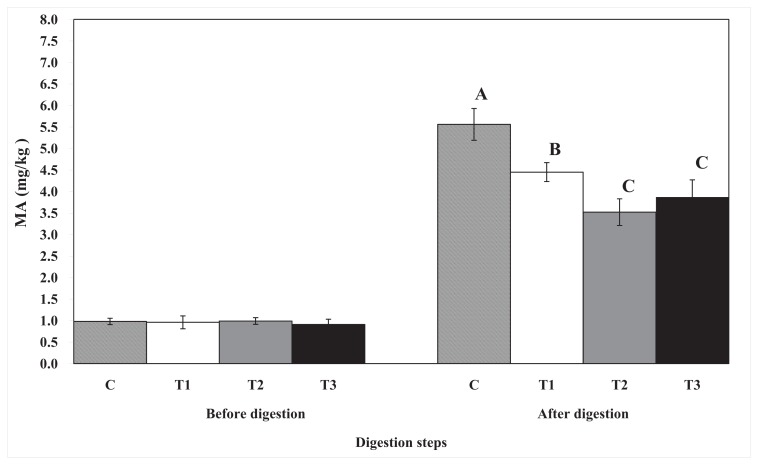
The effect of biopolymer encapsulation of egg yolks on lipid oxidation during *in vitro* human digestion. C: no encapsulation; T1: encapsulation with cellulose; T2: encapsulation with pectin; T3: encapsulation with chitosan. The figure shows the level of total lipid oxidation. MA: Malondialdehyde.

The effects of biopolymer encapsulation on lipid oxidation are shown in Figure 5. The levels of thiobarbituric acid reactive substances (TBARS; used to measure lipid oxidation) in all egg yolk samples increased after *in vitro* human digestion. Egg yolks encapsulated with biopolymers had lower TBARS levels than the control samples. Among the samples encapsulated with biopolymers, pectin- and chitosan-encapsulated egg yolks had lower levels of TBARS than cellulose-encapsulated egg yolks, although TBARS levels in the chitosan- and pectin-encapsulated samples did not differ. The level of TBARS seems to be closely related to the free fatty acid content.

### 2.4. Inhibition of Lipase Activity

**Figure 6:**
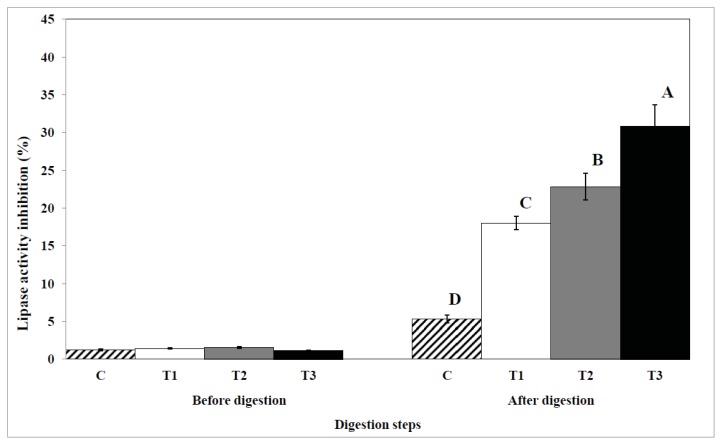
The effect of biopolymer encapsulation of egg yolks on the inhibition of lipase activity during *in vitro* human digestion. C: no encapsulation; T1: encapsulation with cellulose; T2: encapsulation with pectin; T3: encapsulation with chitosan. The inhibition of lipase activity is shown in percentages.

The effects of biopolymer encapsulation on the inhibition of lipase activity are shown in Figure 6. Lipase activity was lower in all egg yolk samples before digestion. However, the inhibition of lipase activity in egg yolks after *in vitro* digestion was significantly increased due to encapsulation with biopolymers. All biopolymers had an inhibitory effect on lipase activity. Cellulose also inhibited lipase activity, although cellulose encapsulation did not have an inhibitory effect on the digestion of total lipids and cholesterol, and on lipid oxidation or free fatty acid content.

### 2.5. Particle Size

**Figure 7:**
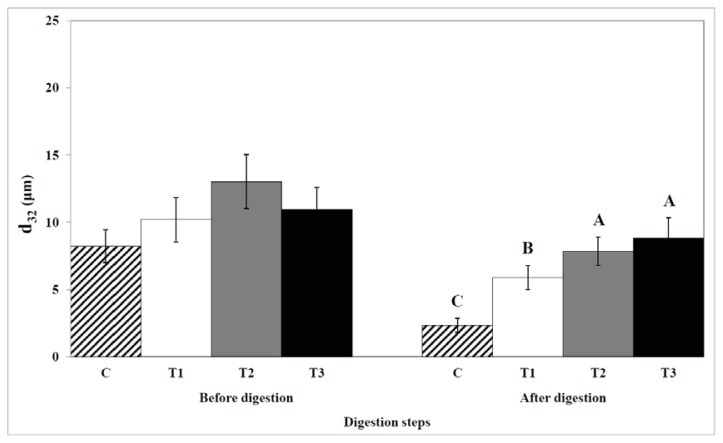
The effect of biopolymer encapsulation of egg yolks on the particle size during *in vitro* human digestion. C: no encapsulation; T1: encapsulation with cellulose; T2: encapsulation with pectin; T3: encapsulation with chitosan. The inhibition of lipase activity is shown in percentages.

The effects of biopolymer encapsulation on the particle size are shown in Figure 7. Particle size was higher in all egg yolk samples before digestion. The particle size in the control and cellulose-encapsulated samples were much smaller than the size of droplets in the chitosan- or pectin-encapsulated samples after digestion.

## 3. Discussion

A number of *in vitro* studies have shown that biopolymers can alter lipid or cholesterol digestion. Dietary fibers can decrease serum cholesterol concentrations by reducing the amount of ingested (exogenous) cholesterol that is adsorbed [10]. Lairon *et al.* reported that some soluble fibers that form viscous solutions drastically reduce the rate of lipid emulsification, with a noticeable decrease in fat lipolysis [11]. Moreover, dietary fiber from various sources can bind to bile acids, mixed micelle components such as monoacylglycerols, free fatty acids, or free cholesterol. This explains the partial disruption of the micellization process, which leads to reduced micellar solubilization of lipid moieties and the reduction in intestinal uptake of lipid moieties and cholesterol [11–13]. In this study, biopolymer-encapsulated egg yolk had lower digestion rates of total lipids and cholesterol in the *in vitro* human digestion model. Moreover, lipase activity was reduced by biopolymer encapsulation. These results indicate that biopolymers, especially pectin and chitosan, easily encapsulate egg yolk lipids and that they can reduce lipase activity. Consequently, lipid and cholesterol digestion was prevented in the *in vitro* human digestion model. In addition, biopolymer encapsulation may interfere with the absorption of total lipids (with cholesterol) into the dialysis tubing membrane (to simulate the villi in the small intestine); or, they may interfere with the ability of lipase to access the lipids contained within them. Beysseriat *et al.* also reported that the ability of dietary fibers to reduce cholesterol digestion and/or absorption through this mechanism depends on their ability to promote droplet aggregation or to adsorb to lipid droplet surfaces [9]. This depends strongly on the electrical charge, molecular weight, and structure of the fibers. For example, cationic chitosan can bind to the surface of anionic lipid droplets that are stabilized by bile salts and/or phospholipids and reduce lipase activity by preventing lipase from coming into contact with the emulsified lipid substrate [9]. The negatively charged lipase may have acted as a bridge between the positively charged chitosan-coated droplets, causing them to clump together, and the interaction of chitosan with lipase may have caused some charge neutralization of the lipid droplets [14].

**Figure 8:**
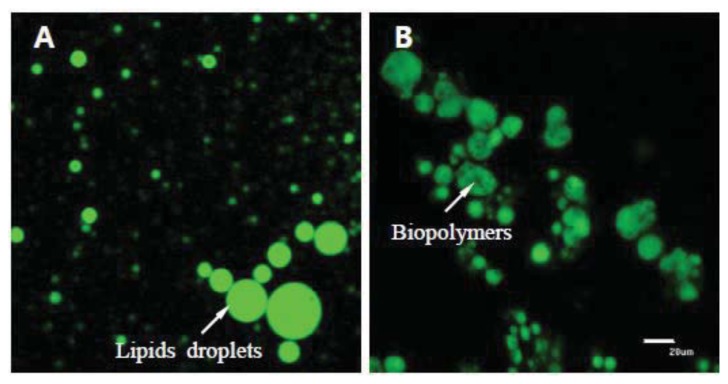
Confocal fluorescence images showing encapsulated lipid with biopolymers in egg yolk. (**A**) Lipid droplets in the egg yolk; (**B**) Lipid droplets with biopolymers in the egg yolk.

Figure 8 shows that biopolymers have the ability to aggregate lipid droplets. This aggregation would decrease the transport rate of enzymes to the lipid surfaces, as well as the transport rate of lipid digestion products present within micelles to the gastrointestinal walls. Thereby, the digestion and absorption rate of total lipids and cholesterol would be reduced.

In an animal study, Fernandez reported that pectin was found to be more effective than guar gum in guinea pigs [15], whereas Moundras *et al.* reported that guar gum was found to be more effective than pectin or gum arabic in rats [16]. In this study, chitosan was found to be more effective for the inhibition of total lipid and cholesterol digestion including the inhibition of lipase activity than other biopolymers during *in vitro* human digestion. This result may be due to the higher electrical charge of chitosan and due to the inhibitory effect of lipase activity. Cellulose had lesser effect on the inhibition of total lipid and cholesterol digestion during *in vitro* human digestion, even though cellulose inhibited lipase activity. This may be because cellulose has a weak electrical charge and, consequently, has no aggregation effect with lipids. In an *in vitro* study, Mun *et al.* reported that chitosan reduced the amount of fatty acids released because lipid droplets were surrounded by cationic chitosan layers or because the lipids droplets were trapped within large chitosan aggregates [17]. They also suggested that chitosan formed a protective layer around the droplets and that it promoted extensive droplet flocculation, both of which inhibited the ability of lipase to interact with the fat inside the droplets. Thus, we assume that the digestion of total lipids and cholesterol would be further reduced in chitosan-encapsulated egg yolks than in those encapsulated with other biopolymers.

Another possible mechanism for the inhibition of lipid digestion is, presumably, the increase in solution viscosity due to biopolymer encapsulation, which causes a reduction in the diffusion of molecules (such as lipase and other enzymes or mixed micelles) and other species in the gastrointestinal tract. As mentioned above, this reduction in diffusion by increasing the solution viscosity would decrease the transport rate of enzymes to the lipid surfaces, thereby reducing the digestion and absorption rates of lipids. Meyer and Doty reported that a high viscosity of the contents of the small intestine may delay lipid digestion, promoting absorption in a more distal part of the small intestine [18]. An increase in solution viscosity is responsible for a decrease in the transit time of ingested food in the gastrointestinal tract. Therefore, an increase in solution viscosity causes a decrease in the transport rate of lipid digestion products present within micelles to the gastrointestinal walls. Khan *et al.* [19] also reported that biopolymers may interfere with the formation of micelles and/or lower the diffusion rate of bile acid and cholesterol-containing micelles through the bolus, consequently diminishing the uptake of cholesterol and bile acids. In our previous study, dietary biopolymers increased the viscosity of the contents of the small intestine [7]. Thus, total lipid and cholesterol digestion in egg yolks can be reduced by biopolymer encapsulation because biopolymers were increased the viscosity of egg yolks in this study (data are not shown). In general, the differences between dietary fibers such as molecular weight and hydrophobicity cause differences in their physicochemical properties such as water solubility, viscosity enhancement, opacity, surface activity, and binding capacity [20]. These differences can cause significant alterations in their effectiveness in reducing cholesterol digestion by interfering with the various physiological processes during digestion and absorption. Yamaguchi *et al.* reported that low molecular weight pectins are more effective in lowering cholesterol than high molecular weight pectins [21]. This has been attributed to the reduction in bile acid binding that occurs when the molecular weight of pectin falls below a critical value [22]. Therefore, the effect of biopolymer encapsulation on total lipid and cholesterol digestion in egg yolk would be largely influenced by the conditions of the biopolymers such as molecular weight, hydrophobicity, viscosity, and pH. Thus, further research is needed to find the most effective biopolymer.

## 4. Experimental Section

### 4.1. Materials

Potassium chloride, sodium sulfate, sodium hydrogen carbonate, hydrogen chloride, potassium phosphate monobasic, magnesium chloride, hexane, trichloroacetic acid, ether, and ethanol were purchased from Fisher Scientific chemical company (Pittsburgh, PA, USA). Cellulose, pectin, chitosan, bicarbonate, potassium thiocyanate, sodium phosphate dibasic, sodium phosphate monobasic, sodium chloride, calcium chloride, ammonium chloride, urea, glucose sigma, glucuronic acid, glucosamine, *α*-amylase, uric acid, mucin, bovine serum albumin, pepsin, pancreatin, lipase, bile salt extraction, Nile red, 5*α*-cholestane, thiobarbituric acid, butylated hydroxyanisole, and phenolphthalein were purchased from Sigma-Aldrich Chemical Co. (St. Louis, MO, USA). Pyridine, bis-[trimethylsilyl] trifluoroacetamide, and trimethylchlorosilane were purchased from Supelco Co. (St. Louis, MO, USA).

### 4.2. Biopolymer Encapsulation Preparation

The experiments were performed by 5 replications from 10 different egg yolk samples. Eggs were purchased from the local market. Chitosan (10 wt%) was dissolved in acetate buffer solutions (100 mM acetic acid: sodium acetate, pH 3.0, 0–150 mM NaCl). Pectin and cellulose (10 wt% each) were dissolved in phosphate buffer solutions (2 M monobasic sodium phosphate and 2 M dibasic sodium phosphate, pH 7.0). These solutions were stirred for 12 h and then mixed for 3 h using a magnetic stirrer. During mixing, 1 mL of Tween 20 (0.1%, pH 7.6) was added dropwise to reduce surface tension and enhance encapsulation formation. The biopolymer encapsulation was prepared by mixing a final volume of 10 wt% biopolymer solution and egg yolk together for 1 h using a bio-homogenizer. The mixture was continuously stirred for 15 min using power ultrasound at a frequency of 10 MHz (final volume: whole egg yolk mixed with 1% biopolymers). This process was aimed at developing a coating layer around the lipophilic egg yolk. Encapsulation of biopolymers and egg yolk was confirmed using confocal microscopy (Figure 8). The molecular characteristics of biopolymers are listed in Table 1.

### 4.3. *In Vitro* Human Digestion

An *in vitro* human digestion model that simulated the mouth, stomach, and small intestine was used in this study, which was a modified version of that described by Versantvoort *et al.* [23].

(1) Initial system: The initial egg yolk samples encapsulated with 1% biopolymers.
(2) Mouth: 10 g of initial egg yolk samples was mixed with 12 mL of simulated saliva solution (pH 6.8) and then stirred for 5 min at 37 °C.
(3) Stomach: 24 mL of simulated gastric juice (pH 2) was then added, and the mixture was stirred for 2 h at 37 °C.
(4) Small intestine: 24 mL of duodenal juice, 12 mL of bile juice, and 2 mL of HCO_3_ solution (pH 6.5 to 7) was then added. The total solution was placed in a 250 mL flask, and then the dialysis tubing (molecular weight cutoff of 50,000, flat width 34 mm, thickness 18 μm, Membrane Filtration Products, Inc. Seguin, TX, USA) containing 10 mL of phosphate buffer (pH 7) was placed in a 250 mL flask, and the mix was stirred for 2 h at 37 °C.

The compositions of the simulated saliva, gastric, duodenal, and bile juices are listed in Table 2. During *in vitro* human digestion, the samples were swirled (60 rpm) on a shaking water bath to simulate the motility of the GI tract (Model 3582, Labline Instruments, Inc., Melrose Park, IL, USA).

### 4.4. Total Lipid and Cholesterol Contents

Total lipids were extracted with chloroform and methanol as described by Folch *et al.* [24]. Cholesterol was determined by the modified method of Russo and others [25]. Briefly, extracted lipid (50 mg) was added into a 50 mL tube with 10 mL of saponification reagent (30% KOH and ethanol with the ratio of 6:94) and 0.5 mL internal standard (2 mg 5 *α*-cholestane/sample), and then capped and incubated for 1 h at 60 °C. After cooling the sample, 8 mL of deionized distilled water and 3 mL hexane were added and mixed thoroughly and allowed to separate. The top layer (hexane layer) was removed and dried in scintillation vials, and 100 μL of bis-[trimethylsilyl]trifluoroacetamide + 1% trimethylchlorosilane (Supelco Co., Bellefonte, PA, USA) and 200 μL of pyridine were added and mixed. The samples were left to set overnight and then analyzed by gas chromatography (Agilent 6890). A ramped oven temperature condition (180 °C for 2.5 min, increased to 230 °C at 2.5 °C/min, then held at 230 °C for 7.5 min) was used. Temperatures of both the inlet and detector were 280 °C. Helium was the carrier gas at linear flow of 1.1 mL/min. Detector (flame ion detector) air, H_2_, and make-up gas (He) flows were 350, 35, and 43 mL/min, respectively.

### 4.5. Free Fatty Acid Content

Free fatty acid content was determined by the modified method of AOAC [26]. Free fatty acid contents were weighed by titrimetry. Briefly, 5 g of sample was weighed into a 50-mL test tube and homogenized with 15 mL of deionized distilled water using a Polytron homogenizer (IKA, Model T25, Staufen, Germany) for 10 s at the highest speed. Then, 2 mL of egg yolk homogenate was transferred to a 300 mL flask, and 100 mL of ether/ethanol solution (ether:ethanol, 1:1, *v*/*v*) was added. Several drops of phenolphthalein were added, and the free fatty acids were titrated with 0.1 M KOH. Free fatty acid (KOH*/*g) = 5.611 × *A* × *F*/Sample weight (g), *A*: volume (mL) of 0.1 M KOH solution; *F*: titer of KOH.

### 4.6. Thiobarbituric Acid-Reactive Substances

TBARS were determined by the modified method of Buege and Aust [27]. Briefly, 5 g of egg yolk sample was weighed into a 50-mL test tube and homogenized with 15 mL of deionized distilled water using a Polytron homogenizer for 10 s at the highest speed (before digestion samples only). Then, 1 mL of egg yolk homogenate was transferred to a disposable test tube (3 × 100 mm), and butylated hydroxyanisole (50 μL, 10%) and thiobarbituric acid/trichloroacetic acid (TBA/TCA) (2 mL) were added. The mixture was vortexed and then incubated in a boiling water bath for 15 min to develop color. The sample was cooled in cold water for 5 min, vortexed again, and centrifuged for 15 min at 2000× *g*. The absorbance of the resulting supernatant solution was determined at 531 nm against a blank containing 1 mL of DDW and 2 mL of TBA/TCA solution. The amounts of TBARS were expressed as milligrams of malondialdehyde (MA) per kilogram of egg yolk samples.

### 4.7. Lipase Activity

Lipase activity was determined by the modified method of Gooda Sahib *et al.* [28]. Porcine lipase was dissolved in 0.01 M Tris-HCl buffer (25 units*/*mL). *In vitro* digested solutions were dissolved in 0.01 M Tris-HCl buffer at different concentrations (7.81–250 ppm). Olive oil (10% *v/v*) was mixed with Arabic gum (10 g) mixture (10% *w/v* in 0.1 M), (1.57 g) Tris-HCl buffer, pH 2, 0.5 M (2.92 g) NaCl, and 20 mM (0.02 g) CaCl_2_ using a homogenizer. Then, 0.2 mL of lipase solution (25 units) was allowed to react with 0.5 mL of *in vitro* digested solution for 30 min at 4 °C. Reconstituted substrate emulsion (2 mL) was then added, and the mixture was incubated for 30 min at 37 °C. The reaction was stopped using acetone and ethanol mixture (1:1/*v:v*) and titrated with 0.02 M NaOH to pH 9.4. Titrations were carried out using an automatic titrator (785 DMP Titrino, Metrohm, Herisau, Switzerland).

### 4.8. Confocal Laser Scanning Microscopy

The total lipid content and biopolymer encapsulation were analyzed through confocal laser scanning microscopy. A confocal scanning fluorescence microscope (Carl Zeiss, LSM 5 Live, GmbH, Jena, Germany) with a 20× objective lens was used to capture confocal images. Nile red (a lipid fluorescent dye) was excited with a 488-nm argon laser line. The fluorescence emitted from the sample was monitored using a fluorescence detector (543 nm) with a pinhole size of 150 μm. The resulting images consisted of 512 × 512 pixels, with a pixel size of 414 nm and a pixel dwell time of 5 s.

### 4.9. Particle Size

The particle size of the sample was measured using a laser light scattering instrument (Mastersizer X, Malvern Instruments Ltd., Malvern, UK). This instrument is based on diffraction of a monochromatic beam of laser light (λ = 632.8 nm) when it is scattered by the droplets in a dilute egg yolk. The instrument measures the angular dependence of the intensity of laser light diffraction and finds the particle size that gives the best fit to the experimental measurements and predictions based on light scattering theory. The mean particle size was reported as the surface-weighted mean diameter, *d*_32_ (= ∑*n*_i_*d*_i_^3^ ∑*n*_i_*d*_i_^2^), where *n*_i_ is the number of particles with diameter *d*_i_.

### 4.10. Statistics

The effect of biopolymer encapsulation on the digestion of total lipids and cholesterol in egg yolks during *in vitro* human digestion were analyzed using SAS software (SAS Inst. Inc., Cary, NC, USA) by the generalized linear model procedure. The Student-Newman-Keuls multiple range test was used to compare differences between means.

## 5. Conclusions

The study results show that encapsulation of egg yolk with biopolymers affected their physical stability and digestibility when they were passed through an *in vitro* human digestion model. The decrease in the diameters of lipid droplets as the droplets moved from the mouth to the stomach and then to the small intestine suggests that encapsulation of egg yolks with biopolymers such as pectin and chitosan can reduce the digestion of total lipids and cholesterol during *in vitro* human digestion. However, encapsulation with various biopolymers had a limited effect on the microstructure changes that occurred during *in vitro* human digestion of lipid droplets in egg yolk. Biopolymers vary widely in their electrical and hydrophobic characteristics, and the postprandial metabolism resulting from the digestion and absorption of available nutrients is a highly complex process involving numerous potential interactions. Therefore, the reduction capacities of total lipids and cholesterol digestion can be expected to vary widely. Thus, further research is needed to understand the effect of biopolymer encapsulation that occurs during *in vitro* human digestion and how biopolymers are associated with the changes in the digestion of total lipids and cholesterol.

## Figures and Tables

**Table 1:** Molecular characteristics of biopolymer ingredients.

Biopolymer type	Electrical charge	Molecular weight	Hydrophobicity
Cellulose	Non-ionic	100 kDa	Low
Pectin	Anionic (–COO^−^) Degree of methylaton: 50	100 kDa	Variable (–CH_3_)
Chitosan	Cationic (NH^3+^) Degree of acetylation: 50	100 kDa	Variable (–COCH_3_)

**Table 2:** Constituents and concentrations of the various synthetic juices of the *in vitro* human digestion model representing fed conditions.

	Saliva	Gastric juice	Duodenal juice	Bile juice
Inorganic components	10 mL KCl 89.6 g/L	15.7 mL NaCl 175.3 g/L	40 mL NaCl 175.3 g/L	30 mL NaCl 175.3 g/L
	10 mL KSCN 20 g/L	3.0 mL NaH_2_PO_4_ 88.8 g/L	40 mL NaHCO_3_ 84.7 g/L	68.3 mL NaHCO_3_ 84.7 g/L
	10 mL NaH_2_PO_4_ 88.8 g/L	9.2 mL KCl 89.6 g/L	10 mL KH_2_PO_4_ 8 g/L	4.2 mL KCl 89.6 g/L
	10 mL NaSO_4_ 57 g/L	18 mL CaCl_2_·2H_2_O 22.2 g/L	6.3 mL KCl 89.6 g/L	150 μL HCl 37% g/g
	1.7 mL NaCl 175.3 g/L	10 mL NH_4_Cl 30.6 g/L	10 mL MgCl_2_ 5 g/L	-
	20 mL NaHCO_3_ 84.7 g/L	6.5 mL HCl 37% g/g	180 μL HCl 37% g/g	20 mL NaHCO_3_ 84.7 g/L

Organic components	8 mL urea 25 g/L	10 mL glucose 65 g/L	4 mL urea 25 g/L	10 mL urea 25 g/L
		10 mL glucuronic acid 2 g/L		
		3.4 mL urea 25 g/L		
		10 mL glucosamine hydrochloride 33 g/L		

Add to mixture of organic + inorganic components	290 mg α-amylase	1 g BSA	9 mL CaCl_2_·2H_2_O 22.2 g/L	10 mL CaCl_2_·2H_2_O 22.2 g/L
	15 mg uric acid	2.5 g pepsin	1 g BSA	1.8 g BSA
	25 mg mucin	3 g mucin	9 g pancreatin	30 g bile
			1.5 g lipase	

pH	6.8 ± 0.2	1.30 ± 0.02	8.1 ± 0.2	8.2 ± 0.2

The inorganic and organic components are augmented to 500 mL with distilled water. If necessary, the pH of the juices was adjusted to the appropriate interval.
